# Supplementary material for: Significant inefficiency in running community health systems: The case of health posts in Southwest Ethiopia
Source: PLoS One. 2021 Feb 19;16(2):e0246559. doi: 10.1371/journal.pone.0246559 (PMC7895414; doi:10.1371/journal.pone.0246559)
Supplement: S1 Table — (DOCX) [file pone.0246559.s001.docx]

**S1 Table. Technical efficiency of health posts, Southwest Ethiopia, 2018**

| **Code** | **Constant returns to scale TE** | **Pure technical efficiency** | **Scale efficiency** | **Returns to scale** |
| --- | --- | --- | --- | --- |
| HP01 | 1 | 1 | 1 | - |
| HP02 | 1 | 1 | 1 | - |
| HP03 | 0.983 | 0.983 | 1 | - |
| HP04 | 0.708 | 0.747 | 0.948 | DRS |
| HP05 | 1 | 1 | 1 | - |
| HP06 | 1 | 1 | 1 | - |
| HP07 | 0.948 | 1 | 0.948 | DRS |
| HP08 | 1 | 1 | 1 | - |
| HP09 | 1 | 1 | 1 | - |
| HP10 | 1 | 1 | 1 | - |
| HP11 | 1 | 1 | 1 | - |
| HP12 | 0.732 | 0.849 | 0.863 | DRS |
| HP13 | 1 | 1 | 1 | - |
| HP14 | 1 | 1 | 1 | - |
| HP15 | 1 | 1 | 1 | - |
| HP16 | 1 | 1 | 1 | - |
| HP17 | 1 | 1 | 1 | - |
| HP18 | 1 | 1 | 1 | - |
| HP19 | 0.727 | 1 | 0.727 | DRS |
| HP20 | 0.897 | 0.92 | 0.975 | IRS |
| HP21 | 0.915 | 1 | 0.915 | DRS |
| HP22 | 0.622 | 0.68 | 0.915 | IRS |
| HP23 | 0.853 | 0.887 | 0.961 | IRS |
| HP24 | 0.873 | 0.887 | 0.984 | DRS |
| HP25 | 0.856 | 1 | 0.856 | DRS |
| HP26 | 0.257 | 0.435 | 0.591 | DRS |
| HP27 | 0.677 | 0.986 | 0.686 | DRS |
| HP28 | 0.714 | 1 | 0.714 | DRS |
| HP29 | 0.597 | 0.652 | 0.915 | DRS |
| HP30 | 0.383 | 0.475 | 0.805 | DRS |
| HP31 | 0.838 | 1 | 0.838 | DRS |
| HP32 | 0.832 | 1 | 0.832 | DRS |
| HP33 | 0.543 | 0.889 | 0.61 | DRS |
| HP34 | 0.713 | 0.992 | 0.719 | DRS |
| HP35 | 0.766 | 0.791 | 0.969 | DRS |
| HP36 | 0.365 | 0.598 | 0.61 | DRS |
| HP37 | 0.671 | 0.671 | 1 | - |
| HP38 | 0.697 | 0.79 | 0.883 | DRS |
| HP39 | 0.645 | 0.916 | 0.704 | DRS |
| HP40 | 0.72 | 0.724 | 0.995 | DRS |
| HP41 | 0.553 | 0.683 | 0.809 | DRS |
| HP42 | 0.33 | 0.431 | 0.765 | DRS |
| HP43 | 0.62 | 0.721 | 0.86 | DRS |
| HP44 | 0.4 | 0.482 | 0.83 | DRS |
| HP45 | 0.603 | 1 | 0.603 | DRS |
| HP46 | 0.303 | 0.442 | 0.686 | DRS |
| HP47 | 0.494 | 0.494 | 1 | - |
| HP48 | 0.419 | 0.63 | 0.666 | DRS |
| HP49 | 0.418 | 0.577 | 0.724 | DRS |
| HP50 | 0.346 | 0.349 | 0.992 | DRS |
| HP51 | 0.456 | 0.456 | 1 | - |
| HP52 | 0.452 | 0.725 | 0.623 | DRS |
| HP53 | 0.469 | 0.741 | 0.633 | DRS |
| HP54 | 0.421 | 0.697 | 0.604 | DRS |
| HP55 | 0.45 | 0.45 | 1 | - |
| HP56 | 0.411 | 0.424 | 0.969 | IRS |
| HP57 | 0.368 | 0.37 | 0.992 | IRS |
| HP58 | 0.382 | 0.415 | 0.919 | DRS |
| HP59 | 0.33 | 0.471 | 0.699 | DRS |
| HP60 | 0.304 | 0.438 | 0.695 | DRS |
| HP61 | 0.254 | 0.556 | 0.458 | DRS |
| HP62 | 0.286 | 0.582 | 0.491 | DRS |
| HP63 | 0.316 | 0.449 | 0.703 | DRS |
| HP64 | 0.257 | 0.35 | 0.736 | DRS |
| HP65 | 0.209 | 0.452 | 0.463 | DRS |
| HP66 | 0.178 | 0.238 | 0.747 | DRS |
|  |  |  |  |  |
| Mean | 0.64 | 0.75 | 0.84 |  |
| SD | 0.27 | 0.24 | 0.16 |  |
| Min | 0.18 | 0.24 | 0.46 |  |
| Max | 1.00 | 1.00 | 1.00 |  |

IRS – Increasing Returns to Scale

DRS – Decreasing Returns to Scale
