# Supplementary material for: Significant inefficiency in running community health systems: The case of health posts in Southwest Ethiopia
Source: PLoS One. 2021 Feb 19;16(2):e0246559. doi: 10.1371/journal.pone.0246559 (PMC7895414; doi:10.1371/journal.pone.0246559)
Supplement: S2 Table — (DOCX) [file pone.0246559.s002.docx]

**S2 Table. Summary of targets and potential increase in outputs of health posts, Southwest Ethiopia, 2018**

| **HPs** | **Health Education Sessions** | | **ANC** | | **FP service** | | **Diarrhea treated** | | **HH visit** | | **Malaria treated** | | **Children Immunization** | | **Referral** | |
| --- | --- | --- | --- | --- | --- | --- | --- | --- | --- | --- | --- | --- | --- | --- | --- | --- |
|  | **Target** | **Po Inc*** | **Target** | **Po Inc*** | **Target** | **Po Inc*** | **Target** | **Po Inc*** | **Target** | **Po Inc*** | **Target** | **Po Inc*** | **Target** | **Po Inc*** | **Target** | **Po Inc*** |
| HP01 | 4 | 0 | 120 | 0 | 265 | 0 | 134 | 0 | 930 | 0 | 10 | 0 | 120 | 0 | 2 | 0 |
| HP02 | 36 | 0 | 210 | 0 | 2753 | 0 | 73 | 0 | 650 | 0 | 0 | 0 | 182 | 0 | 39 | 0 |
| HP03 | 60 | 12 | 226 | 4 | 633 | 313 | 134 | 2 | 874 | 34 | 30 | 28 | 220 | 4 | 4 | 1 |
| HP04 | 20 | 5 | 149 | 101 | 733 | 186 | 158 | 40 | 851 | 444 | 0 | 0 | 297 | 135 | 13 | 3 |
| HP05 | 18 | 0 | 53 | 0 | 1155 | 0 | 17 | 0 | 2304 | 0 | 0 | 0 | 148 | 0 | 6 | 0 |
| HP06 | 84 | 0 | 156 | 0 | 662 | 0 | 60 | 0 | 302 | 0 | 12 | 0 | 112 | 0 | 4 | 0 |
| HP07 | 12 | 0 | 94 | 0 | 1480 | 0 | 182 | 0 | 1320 | 0 | 0 | 0 | 208 | 0 | 528 | 0 |
| HP08 | 12 | 0 | 141 | 0 | 1638 | 0 | 223 | 0 | 792 | 0 | 0 | 0 | 183 | 0 | 611 | 0 |
| HP09 | 8 | 0 | 97 | 0 | 480 | 0 | 150 | 0 | 1920 | 0 | 0 | 0 | 102 | 0 | 1080 | 0 |
| HP10 | 7 | 0 | 144 | 0 | 720 | 0 | 528 | 0 | 720 | 0 | 0 | 0 | 1080 | 0 | 192 | 0 |
| HP11 | 72 | 0 | 108 | 0 | 660 | 0 | 204 | 0 | 684 | 0 | 11 | 0 | 136 | 0 | 0 | 0 |
| HP12 | 58 | 34 | 231 | 35 | 639 | 97 | 25 | 8 | 1096 | 166 | 32 | 17 | 451 | 253 | 6 | 1 |
| HP13 | 5 | 0 | 231 | 0 | 893 | 0 | 186 | 0 | 931 | 0 | 14 | 0 | 920 | 0 | 22 | 0 |
| HP14 | 64 | 0 | 192 | 0 | 876 | 0 | 100 | 0 | 3000 | 0 | 30 | 0 | 480 | 0 | 25 | 0 |
| HP15 | 40 | 0 | 432 | 0 | 480 | 0 | 36 | 0 | 240 | 0 | 60 | 0 | 240 | 0 | 0 | 0 |
| HP16 | 79 | 0 | 48 | 0 | 442 | 0 | 6 | 0 | 1945 | 0 | 12 | 0 | 692 | 0 | 3 | 0 |
| HP17 | 61 | 0 | 48 | 0 | 329 | 0 | 18 | 0 | 1548 | 0 | 0 | 0 | 547 | 0 | 3 | 0 |
| HP18 | 41 | 0 | 48 | 0 | 242 | 0 | 18 | 0 | 1130 | 0 | 0 | 0 | 368 | 0 | 2 | 0 |
| HP19 | 18 | 0 | 332 | 0 | 866 | 0 | 16 | 0 | 550 | 0 | 70 | 0 | 154 | 0 | 4 | 0 |
| HP20 | 64 | 51 | 192 | 146 | 876 | 70 | 100 | 88 | 3000 | 1650 | 0 | 0 | 480 | 284 | 25 | 19 |
| HP21 | 90 | 0 | 72 | 0 | 386 | 0 | 11 | 0 | 1752 | 0 | 0 | 0 | 618 | 0 | 2 | 0 |
| HP22 | 53 | 45 | 305 | 225 | 690 | 300 | 70 | 40 | 1701 | 1301 | 44 | 14 | 367 | 257 | 13 | 4 |
| HP23 | 62 | 18 | 207 | 23 | 700 | 386 | 131 | 15 | 1424 | 618 | 28 | 22 | 282 | 112 | 9 | 1 |
| HP24 | 79 | 9 | 54 | 6 | 464 | 52 | 18 | 5 | 1855 | 376 | 11 | 8 | 654 | 74 | 3 | 2 |
| HP25 | 124 | 0 | 280 | 0 | 25 | 0 | 252 | 0 | 69 | 0 | 107 | 0 | 8 | 0 | 5 | 0 |
| HP26 | 60 | 34 | 114 | 72 | 1292 | 730 | 62 | 35 | 1391 | 1041 | 0 | 0 | 526 | 297 | 27 | 23 |
| HP27 | 97 | 1 | 209 | 113 | 376 | 280 | 203 | 3 | 699 | 603 | 62 | 38 | 122 | 2 | 9 | 0 |
| HP28 | 95 | 0 | 41 | 0 | 1122 | 0 | 25 | 0 | 2000 | 0 | 0 | 0 | 295 | 0 | 2 | 0 |
| HP29 | 33 | 13 | 268 | 93 | 1845 | 642 | 80 | 28 | 606 | 256 | 0 | 0 | 311 | 108 | 32 | 11 |
| HP30 | 38 | 20 | 194 | 102 | 1617 | 849 | 109 | 73 | 1205 | 735 | 0 | 0 | 505 | 265 | 30 | 23 |
| HP31 | 68 | 0 | 107 | 0 | 486 | 0 | 93 | 0 | 3400 | 0 | 0 | 0 | 181 | 0 | 12 | 0 |
| HP32 | 24 | 0 | 557 | 0 | 607 | 0 | 18 | 0 | 2264 | 0 | 0 | 0 | 164 | 0 | 4 | 0 |
| HP33 | 20 | 17 | 282 | 31 | 810 | 90 | 169 | 19 | 1047 | 116 | 23 | 3 | 680 | 429 | 35 | 28 |
| HP34 | 58 | 43 | 196 | 83 | 1246 | 10 | 95 | 75 | 2537 | 21 | 0 | 0 | 421 | 207 | 28 | 23 |
| HP35 | 37 | 13 | 122 | 31 | 1429 | 299 | 90 | 19 | 1655 | 346 | 0 | 0 | 229 | 52 | 22 | 18 |
| HP36 | 40 | 16 | 408 | 164 | 653 | 263 | 99 | 40 | 889 | 569 | 38 | 31 | 278 | 112 | 20 | 15 |
| HP37 | 72 | 24 | 151 | 63 | 743 | 523 | 133 | 117 | 1487 | 1303 | 0 | 0 | 262 | 86 | 10 | 8 |
| HP38 | 53 | 48 | 90 | 19 | 518 | 274 | 177 | 37 | 1177 | 247 | 0 | 0 | 530 | 464 | 39 | 23 |
| HP39 | 73 | 49 | 142 | 12 | 638 | 54 | 31 | 3 | 1419 | 119 | 0 | 0 | 541 | 439 | 12 | 1 |
| HP40 | 62 | 44 | 105 | 29 | 824 | 228 | 21 | 13 | 1729 | 478 | 0 | 0 | 516 | 313 | 8 | 6 |
| HP41 | 46 | 34 | 162 | 96 | 777 | 246 | 116 | 37 | 2635 | 835 | 0 | 0 | 360 | 263 | 351 | 111 |
| HP42 | 38 | 35 | 304 | 173 | 1127 | 641 | 118 | 67 | 1901 | 1081 | 13 | 8 | 404 | 307 | 42 | 24 |
| HP43 | 39 | 36 | 197 | 55 | 752 | 412 | 229 | 64 | 1291 | 360 | 19 | 13 | 595 | 270 | 54 | 30 |
| HP44 | 46 | 24 | 191 | 99 | 1579 | 818 | 75 | 65 | 1018 | 906 | 0 | 0 | 446 | 231 | 0 | 0 |
| HP45 | 86 | 0 | 216 | 0 | 960 | 0 | 96 | 0 | 312 | 0 | 11 | 0 | 184 | 0 | 7 | 0 |
| HP46 | 36 | 34 | 371 | 207 | 566 | 427 | 58 | 45 | 815 | 455 | 39 | 38 | 396 | 221 | 11 | 6 |
| HP47 | 51 | 39 | 320 | 162 | 582 | 392 | 85 | 43 | 759 | 384 | 44 | 42 | 252 | 144 | 4 | 3 |
| HP48 | 87 | 32 | 75 | 34 | 1130 | 418 | 41 | 32 | 2142 | 792 | 0 | 0 | 326 | 192 | 0 | 0 |
| HP49 | 26 | 14 | 228 | 217 | 1969 | 832 | 119 | 73 | 686 | 472 | 14 | 6 | 372 | 157 | 43 | 32 |
| HP50 | 48 | 35 | 198 | 186 | 1794 | 1167 | 94 | 61 | 1789 | 1680 | 14 | 10 | 332 | 216 | 53 | 50 |
| HP51 | 59 | 47 | 243 | 132 | 614 | 362 | 127 | 69 | 769 | 418 | 0 | 0 | 215 | 195 | 0 | 0 |
| HP52 | 23 | 18 | 263 | 72 | 718 | 359 | 270 | 74 | 1284 | 353 | 15 | 4 | 709 | 646 | 88 | 77 |
| HP53 | 84 | 22 | 183 | 47 | 1082 | 280 | 82 | 43 | 757 | 649 | 0 | 0 | 217 | 56 | 9 | 2 |
| HP54 | 72 | 22 | 293 | 89 | 452 | 177 | 138 | 42 | 1319 | 399 | 50 | 47 | 304 | 92 | 18 | 12 |
| HP55 | 64 | 53 | 192 | 127 | 876 | 616 | 100 | 89 | 3000 | 1649 | 0 | 0 | 480 | 384 | 25 | 23 |
| HP56 | 61 | 49 | 217 | 125 | 748 | 516 | 113 | 65 | 1911 | 1664 | 0 | 0 | 351 | 224 | 14 | 8 |
| HP57 | 50 | 38 | 201 | 193 | 1815 | 1143 | 87 | 78 | 1825 | 1663 | 0 | 0 | 331 | 270 | 32 | 30 |
| HP58 | 41 | 29 | 279 | 163 | 1617 | 945 | 80 | 59 | 904 | 757 | 25 | 24 | 279 | 163 | 0 | 0 |
| HP59 | 66 | 35 | 233 | 123 | 1237 | 654 | 112 | 70 | 1612 | 1295 | 0 | 0 | 280 | 148 | 23 | 21 |
| HP60 | 41 | 31 | 289 | 201 | 1022 | 575 | 73 | 41 | 880 | 495 | 55 | 31 | 201 | 113 | 37 | 21 |
| HP61 | 101 | 45 | 119 | 53 | 869 | 386 | 94 | 55 | 1374 | 916 | 0 | 0 | 205 | 97 | 7 | 3 |
| HP62 | 107 | 45 | 143 | 60 | 663 | 277 | 121 | 94 | 1181 | 752 | 0 | 0 | 173 | 89 | 9 | 4 |
| HP63 | 45 | 41 | 272 | 192 | 1138 | 627 | 39 | 22 | 779 | 429 | 33 | 18 | 312 | 173 | 24 | 13 |
| HP64 | 35 | 23 | 291 | 189 | 869 | 565 | 85 | 66 | 720 | 504 | 30 | 28 | 503 | 327 | 12 | 8 |
| HP65 | 53 | 29 | 268 | 147 | 1170 | 641 | 147 | 140 | 908 | 558 | 0 | 0 | 345 | 189 | 21 | 20 |
| HP66 | 34 | 28 | 302 | 230 | 1284 | 978 | 122 | 93 | 1225 | 933 | 18 | 15 | 340 | 259 | 81 | 73 |
|  |  |  |  |  |  |  |  |  |  |  |  |  |  |  |  |  |
| **Mean** | 52 | 20 | 200 | 72 | 920 | 304 | 107 | 34 | 1347 | 467 | 15 | 7 | 356 | 141 | 58 | 12 |
| **SD** | 27 | 18 | 101 | 75 | 488 | 322 | 81 | 35 | 720 | 508 | 22 | 12 | 199 | 146 | 166 | 20 |
| **Sum** | 3441 | 1335 | 13207 | 4725 | 60699 | 20096 | 7073 | 2244 | 88888 | 30821 | 975 | 446 | 23524 | 9321 | 3859 | 784 |
| **Min** | 4 | 0 | 41 | 0 | 25 | 0 | 6 | 0 | 69 | 0 | 0 | 0 | 8 | 0 | 0 | 0 |
| **Max** | 124 | 53 | 557 | 230 | 2753 | 1167 | 528 | 140 | 3400 | 1680 | 107 | 47 | 1080 | 646 | 1080 | 111 |

*Potential increase
