# Supplementary material for: Significant inefficiency in running community health systems: The case of health posts in Southwest Ethiopia
Source: PLoS One. 2021 Feb 19;16(2):e0246559. doi: 10.1371/journal.pone.0246559 (PMC7895414; doi:10.1371/journal.pone.0246559)
Supplement: S3 Table — (DOCX) [file pone.0246559.s003.docx]

**S3 Table. Potential output increase in inefficient health posts, Southwest Ethiopia, 2018**

|  | Health Education Sessions | ANC | FP service | Diarrhea treated | HH visit | Malaria treated | Children Immunization | Referral |
| --- | --- | --- | --- | --- | --- | --- | --- | --- |
| Mean | 26 | 91 | 386 | 43 | 593 | 9 | 179 | 15 |
| SD | 17 | 73 | 316 | 34 | 503 | 13 | 142 | 22 |
| Sum | 1335 | 4725 | 20096 | 2244 | 30821 | 446 | 9321 | 784 |
| Min | 0 | 0 | 0 | 0 | 0 | 0 | 0 | 0 |
| Max | 53 | 230 | 1167 | 140 | 1680 | 47 | 646 | 111 |
