# Supplementary material for: Significant inefficiency in running community health systems: The case of health posts in Southwest Ethiopia
Source: PLoS One. 2021 Feb 19;16(2):e0246559. doi: 10.1371/journal.pone.0246559 (PMC7895414; doi:10.1371/journal.pone.0246559)
Supplement: Attachment — Submitted filename: Response to reviewers.docx [file pone.0246559.s005.docx]

PONE-D-19-28271
Significant inefficiency in running community health systems: the case of the Health Extension Program in Southwest Ethiopia
PLOS ONE

Dear Dr. Yitbarek,

Thank you for submitting your manuscript to PLOS ONE. After careful consideration, we feel that it has merit but does not fully meet PLOS ONE’s publication criteria as it currently stands. Therefore, we invite you to submit a revised version of the manuscript that addresses the points raised during the review process.

The manuscript has been evaluated by three reviewers, their comments are available below.

The reviewers have raised a number of concerns, regarding the reporting of methodological aspects of the study such as insufficient explanation of the statistical analysis used. In addition, they request a careful consideration of the terminology used to ensure that this is sufficiently defined. Please also note that your manuscript requires copyediting.

Please note that reviewer #2 has also suggested the inclusion of additional references but we do not require you to include these in your next revision.

Could you please carefully revise the manuscript to address all comments raised.

Please submit your revised manuscript by Nov 14 2020 11:59PM. If you will need more time than this to complete your revisions, please reply to this message or contact the journal office at plosone@plos.org. When you're ready to submit your revision, log on to https://www.editorialmanager.com/pone/ and select the 'Submissions Needing Revision' folder to locate your manuscript file.

Please include the following items when submitting your revised manuscript:

- A rebuttal letter that responds to each point raised by the academic editor and reviewer(s). You should upload this letter as a separate file labeled 'Response to Reviewers'.
- A marked-up copy of your manuscript that highlights changes made to the original version. You should upload this as a separate file labeled 'Revised Manuscript with Track Changes'.
- An unmarked version of your revised paper without tracked changes. You should upload this as a separate file labeled 'Manuscript'.

If you would like to make changes to your financial disclosure, please include your updated statement in your cover letter. Guidelines for resubmitting your figure files are available below the reviewer comments at the end of this letter.

If applicable, we recommend that you deposit your laboratory protocols in protocols.io to enhance the reproducibility of your results. Protocols.io assigns your protocol its own identifier (DOI) so that it can be cited independently in the future. For instructions see: <http://journals.plos.org/plosone/s/submission-guidelines#loc-laboratory-protocols>

We look forward to receiving your revised manuscript.

Kind regards,

Sara Fuentes Perez, PhD
Staff Editor
PLOS ONE

**Dear Editors,**

Thank you very much for giving me the chance to revise our manuscript. I have critically read all the comments raised by the editor and reviewers, and revised the manuscript accordingly. Moreover, we have provided responses to editor’s and reviewers comments in a point by point fassion.

Journal Requirements:

When submitting your revision, we need you to address these additional requirements.

1. Please ensure that your manuscript meets PLOS ONE's style requirements, including those for file naming. The PLOS ONE style templates can be found at

https://journals.plos.org/plosone/s/file?id=wjVg/PLOSOne_formatting_sample_main_body.pdf and

https://journals.plos.org/plosone/s/file?id=ba62/PLOSOne_formatting_sample_title_authors_affiliations.pdf

Response: Thank you for the reminder. We have kept the guidelines for the revised copy.

2. Please refer to any post-hoc corrections to correct for multiple comparisons during your statistical analyses. If these were not performed please justify the reasons. Please refer to our statistical reporting guidelines for assistance (https://journals.plos.org/plosone/s/submission-guidelines.#loc-statistical-reporting).

Response: Thank you. We were not requested to perform any corrections to the statistical analysis by the reviewers and the editor.

3. Thank you for stating the following in the Acknowledgments Section of your manuscript:

"We would like to thank Jimma University for funding this study. Moreover, our gratitude goes to data collectors, supervisors and respondents."

We note that you have provided funding information that is not currently declared in your Funding Statement. However, funding information should not appear in the Acknowledgments section or other areas of your manuscript. We will only publish funding information present in the Funding Statement section of the online submission form.

Please remove any funding-related text from the manuscript and let us know how you would like to update your Funding Statement. Currently, your Funding Statement reads as follows:

"The funders had no role in study design, data collection and analysis, decision to publish, or preparation of the manuscript."

Please include your amended statements within your cover letter; we will change the online submission form on your behalf.

Response: Thank you very much for the suggestion. The funder of this project is Jimma University. Although the funders support the study they had no role in study design, data collection and analysis, decision to publish, or preparation of the manuscript. We have put this in the online submission system. The acknowledgement was corrected in the revised copy.

4. Your ethics statement should only appear in the Methods section of your manuscript. If your ethics statement is written in any section besides the Methods, please move it to the Methods section and delete it from any other section. Please ensure that your ethics statement is included in your manuscript, as the ethics statement entered into the online submission form will not be published alongside your manuscript.

Response: Thanks, corrected in the revised copy.

5. Please include captions for your Supporting Information files at the end of your manuscript, and update any in-text citations to match accordingly. Please see our Supporting Information guidelines for more information: http://journals.plos.org/plosone/s/supporting-information.

Response: Thank you very much. The captions are now included at the final page of the main manuscript document.

[Note: HTML markup is below. Please do not edit.]

Reviewers' comments:

Reviewer's Responses to Questions

**Comments to the Author**

1. Is the manuscript technically sound, and do the data support the conclusions?

The manuscript must describe a technically sound piece of scientific research with data that supports the conclusions. Experiments must have been conducted rigorously, with appropriate controls, replication, and sample sizes. The conclusions must be drawn appropriately based on the data presented.

- Reviewer #1: Partly
- Reviewer #2: Yes
- Reviewer #3: Partly

2. Has the statistical analysis been performed appropriately and rigorously?

- Reviewer #1: I Don't Know
- Reviewer #2: Yes
- Reviewer #3: No

3. Have the authors made all data underlying the findings in their manuscript fully available?

The [PLOS Data policy](http://www.plosone.org/static/policies.action#sharing) requires authors to make all data underlying the findings described in their manuscript fully available without restriction, with rare exception (please refer to the Data Availability Statement in the manuscript PDF file). The data should be provided as part of the manuscript or its supporting information, or deposited to a public repository. For example, in addition to summary statistics, the data points behind means, medians and variance measures should be available. If there are restrictions on publicly sharing data—e.g. participant privacy or use of data from a third party—those must be specified.

- Reviewer #1: Yes
- Reviewer #2: Yes
- Reviewer #3: No

4. Is the manuscript presented in an intelligible fashion and written in standard English?

PLOS ONE does not copyedit accepted manuscripts, so the language in submitted articles must be clear, correct, and unambiguous. Any typographical or grammatical errors should be corrected at revision, so please note any specific errors here.

- Reviewer #1: No
- Reviewer #2: Yes
- Reviewer #3: No

5. Review Comments to the Author

Please use the space provided to explain your answers to the questions above. You may also include additional comments for the author, including concerns about dual publication, research ethics, or publication ethics. (Please upload your review as an attachment if it exceeds 20,000 characters)

**Reviewer #1: General comments**

**Dear reviewer,**

Thank you very much for raising very important issues for the improvement of our work. That is really helpful. We have revised the manuscript based on your valuable suggestions. Bellow I have given responses for all the raised questions in a point by point fashion.

This paper, if it is going to be accepted for publication, will need some major revisions. There are as follows:

1. A clear definition of “technical efficiency” and how it is measured. After reading the paper, I had no clear sense of what this term actually referred to. Why is this important, and what practical steps could be taken to improve technical efficiency?

Response: Thank you for the relevant suggestion. We now included description about technical efficiency in the introduction section of the revised manuscript.

2. Although the title of the paper is on the technical efficiency of the Health Extension Program, the text focuses on health posts rather than the HEWs themselves. But since the measures of technical efficiency focus on the HEWs and their work outside of the health post as well as within it (including household visits and services provided individually by HEWs in the community), the narrative should as well.

Response: you are right the paper is on technical efficiency of health extension program. And we have used health posts as decision making units.

Health extension workers (HEWs) are community health workers who give service to the community in different approaches including outreach services, home-to-home services and based in the health post. A health post is staffed with averagely 2 – 4 HEWs whose primary role is implementing the health extension program. There are also resources relevant for health extension program in the health posts.

Therefore, we have taken a health post as a decision making unit (DMU) (unit of analysis in this case) instead of individual HEW, because the health extension program cannot function without considering HEWs and other relevant resources.

Specific major comments

1. We need a definition of “Data Development Analysis”.

Response: thank you for the insightful suggestion. We now included the definition in the introduction briefly and in the method of analysis sub-section in detail in the revise manuscript.

2. The Tobit regression methodology needs explanation for the uninformed reader (which will be 99% of the readers of this article”.

Response: Thank you for the suggestion. We have written explanation in the method of analysis section of the revised manuscript.

3. Need to explain Tools for Assessing the Operationality of District Health Systems (p. 7, line 114-5).

Response: Tools for Assessing the Operationality of District Health Systems is a document that states how we should take sample size in a district and health facility based studies. The suggestions were given by WHO. It has suggestions of number of health facilities and districts to be sampled based on the total number of districts and health service organizations (population) in the study area. The sampling of this study was performed based on the guideline.

4. Need explanation of Data Envelopment Analysis (p. 8, line 133).

Response: Thank you. Detailed explanation was given under the method of analysis sub-section of the revised manuscript.

**Specific minor comments**

1. P. 2 line 31: “severely” is not an appropriate word here.

Response: Thank you. Corrected in the revised copy.

2. P. 4 line 56: Ref 13 is the wrong ref.

Response: Thank you, corrected in the revised copy.

3. P. 5 line 62: Ref 14 is outdated. Here is a suggestion for an up-to-date reference: Perry H. Health for the People: National Community Health Programs from Afghanistan to Zimbabwe. 2020. <https://pdf.usaid.gov/pdf_docs/PA00WKKN.pdf>.

Response: Thank you very much. We have replaced the reference in the revised manuscript.

4. P. 6, line 100: Should define the date at which that exchange rate was measured.

Response: Thank you for the suggestion. We have used the August 2018 exchange rate. Included in the revised copy.

5. P. 8, line 137: You use the term TE here but not consistently for technical efficiency. There should be consistent use throughout the paper.

Response: Thank you for the suggestion. Corrected in the entire document except in the abstract and titles of the revised manuscript.

6. P. 10, line 154: Need to define what you mean by “near a health center”.

Response: Thank you, it is to mean “in a kebele where health center is available”. Described in the revised copy.

7. P. 10, line 144: Are you referring to how long the health post has been in operation or to how long the HEWs have been working there?

Response: in the specified line, we have described about the nature of the efficiency scores. The efficiency scores take values between 0 and 1. To come up with these scores we considered inputs like number of health extension workers working in the specific health post and non-salary recurrent expenditures.

8. P. 10, line 160: There is no need for 2 decimal places anywhere in the paper.

Response: thank you. We have changed to one decimal place except the monetary values and some very low p-values and confidence intervals, in the revised copy.

9. P. 10, line 161: Your bring in the term “scale efficiency” without defining it.

Response: now it is defined well under DEA conceptual framework section of the revised manuscript.

10. P. 11, line 166: You bring in the terms increasing and decreasing returns to scale without defining them.

Response: now it is defined well under DEA conceptual framework section of the revised manuscript.

11. P. 12, line 180: Are you referring to the average spent by each health post?

Response: At the point of the study, health posts are expected to be staffed with an average of 2 HEWs. However, practically there were some health posts that have more than 2. Therefore, 143 HEWs for 66 health posts is slightly above two.

HEWs and other resources were seen as a separate input variables in the study.

12. P. 14, lines 209-211: This sentence is unclear.

Response: We have added sentences before the mentioned sentence. Now it seems better for understanding.

13. P. 15, line 220: The word “sever” is inappropriate and also misspelled.

Response: Thank you, corrected in the revised copy.

14. P. 15, lines 238-242: Not clear how this was computed.

Response: Thank you for this important question. Potential output increase was described in table 2. The description in the mentioned page and line is the proportion of potential output increase taking the actual outputs as a denominator.

15. P. 15, lines 246-248: Good point. But, there is a word missing after “significant”.

Response: Thank you, corrected in the revised copy.

16. P. 15, line 251: Should this be “decrease” rather than “increase?

Response: Thank you, corrected in the revised copy.

**Reviewer #2**

**Dear reviewer,**

Thank you very much for the suggestions and reference materials you provided to me. They are really helpful.

Response: However, most of them are macro-economic analyses, despite the micro-economic nature of our paper. Our paper studied the technical efficiency (a process measure) of Ethiopian community health service under the primary health care. We therefore, added other relevant references that complies with our manuscript.

Reviewer #2: This is a decent contribution on inefficiency in running community health systems in the example of Health Extension Program in Ethiopia.

It fills certain knowledge gap and is worthy of publishing. Yet the evidence base should be signficantly expanded.

OECD academic sources dominate alongside with few national ones. Much more LMICs and EMerging Markets documented evidence should be added to increase diversity and reliability of the claims in the text.

Thus I warmly recommend introduction of some of the following sources listed beneath:

- Jakovljevic, M., Timofeyev, Y., Ranabhat, C. et al. Real GDP growth rates and healthcare spending – comparison between the G7 and the EM7 countries. Global Health 16, 64 (2020). <https://doi.org/10.1186/s12992-020-00590-3>
- Jakovljevic, M., Matter-Walstra, K., Sugahara, T. et al. Cost-effectiveness and resource allocation (CERA) 18 years of evolution: maturity of adulthood and promise beyond tomorrow. Cost Eff Resour Alloc 18, 15 (2020). <https://doi.org/10.1186/s12962-020-00210-2>
- Jakovljevic, M., Potapchik, E., Popovich, L., Barik, D., & Getzen, T. E. (2017). Evolving health expenditure landscape of the BRICS nations and projections to 2025. Health economics, 26(7), 844-852.
- Jakovljevic, M., Jakab, M., Gerdtham, U., McDaid, D., Ogura, S., Varavikova, E., ... & Getzen, T. E. (2019). Comparative financing analysis and political economy of noncommunicable diseases. Journal of medical economics, 22(8), 722-727.
- Rancic, N., & Jakovljevic, M. M. (2016). Long term health spending alongside population aging in N-11 emerging nations. East Eur Bus Econ J, 2(1), 2-26.
- Jakovljevic, M., & Getzen, T. E. (2016). Growth of global health spending share in low and middle income countries. Frontiers in pharmacology, 7, 21. <https://www.frontiersin.org/articles/10.3389/fphar.2016.00021/full>
- Dieleman, J. L., Campbell, M., Chapin, A., Eldrenkamp, E., Fan, V. Y., Haakenstad, A., ... & Reynolds, A. (2017). Future and potential spending on health 2015–40: development assistance for health, and government, prepaid private, and out-of-pocket health spending in 184 countries. The Lancet, 389(10083), 2005-2030.
- Jakovljevic, M. B. (2014). The key role of the leading emerging BRIC markets in the future of global health care. Serbian Journal of Experimental and Clinical Research, 15(3), 139-143.
- Jakovljevic, M. B. (2015). BRIC’s growing share of global health spending and their diverging pathways. Frontiers in public health, 3, 135.https://www.frontiersin.org/articles/10.3389/fpubh.2015.00135/full
- Jakovljevic, M., Groot, W., & Souliotis, K. (2016). Health care financing and affordability in the emerging global markets. Frontiers in public health, 4, 2. <https://www.frontiersin.org/articles/10.3389/fpubh.2016.00002/full>

Conditional to adopting at least several of these remarks, I am willing to review the revised manuscript assuming its maturity for publishing.

**Reviewer #3**

**Dear reviewer,**

Thank you very much for you time reviewing our manuscript and your valuable inputs. We made changes to the manuscript based on your suggestions. Bellow we have given responses to all the raised questions.

Reviewer #3: General comment: The paper requires language polishing because it is hard to read.

**Response**: Thank you very much for the suggestion. We did a copy edit to polish the grammatical errors and write-up issues.

For instance: ...and changed
100 in to US dollar ...

**Response**: Thank you. Corrected in the revised copy.

Statistical analysis:
Data Envelopment Analysis (DEA) was performed - Add a citation for this.

**Response**: Thank you very much. We put citation and further explanation for the model.

used to estimate
135 the technical efficiency scores - Describe these scores in detail.

**Response**: Thank you very much. We have given sufficient explanation about DEA model and TE scores under the “DEA conceptual framework” sub-title.

Tobit regression model - Add a citation and a discussion of the model.

**Response**: Thank you for the insightful suggestion. We have put a sufficient explanation about the Tobit model with the relevant citation in the revised copy.

Statistical significance was declared with p-values less than 0.05, and the 95% confidence
147 interval of coefficients. - What you mean is:
Statistical significance was declared for a significance level of 0.05, and the 95% confidence
147 interval of coefficients.

Add citation for this:
<https://www.mdpi.com/2504-4990/1/3/54>

**Response**: Thank you very much for the suggestion. We made changes and added the citation in the revised copy according to the suggestion in the revised manuscript.

Table 4: From the text, the covariates used for the regression analysis are unclear. Provide this information explicitely in the table and the main text.

**Response**: Thank you for the suggestion. We put further description of the variables in the “Methods” section, under “Variables” sub heading in the revised copy.
