# Supplementary material for: Significant inefficiency in running community health systems: The case of health posts in Southwest Ethiopia
Source: PLoS One. 2021 Feb 19;16(2):e0246559. doi: 10.1371/journal.pone.0246559 (PMC7895414; doi:10.1371/journal.pone.0246559)
Supplement: Attachment — Submitted filename: Response to reviewers.docx [file pone.0246559.s006.docx]

PONE-D-19-28271R1
Significant inefficiency in running community health systems: the case of the Health Extension Program in Southwest Ethiopia
PLOS ONE

Dear Dr. Yitbarek,

Thank you for submitting your manuscript to PLOS ONE. After careful consideration, we feel that it has merit but does not fully meet PLOS ONE’s publication criteria as it currently stands. Therefore, we invite you to submit a revised version of the manuscript that addresses the points raised during the review process.

Please submit your revised manuscript by Jan 14 2021 11:59PM. If you will need more time than this to complete your revisions, please reply to this message or contact the journal office at plosone@plos.org. When you're ready to submit your revision, log on to https://www.editorialmanager.com/pone/ and select the 'Submissions Needing Revision' folder to locate your manuscript file.

Please include the following items when submitting your revised manuscript:

- A rebuttal letter that responds to each point raised by the academic editor and reviewer(s). You should upload this letter as a separate file labeled 'Response to Reviewers'.
- A marked-up copy of your manuscript that highlights changes made to the original version. You should upload this as a separate file labeled 'Revised Manuscript with Track Changes'.
- An unmarked version of your revised paper without tracked changes. You should upload this as a separate file labeled 'Manuscript'.

If you would like to make changes to your financial disclosure, please include your updated statement in your cover letter. Guidelines for resubmitting your figure files are available below the reviewer comments at the end of this letter.

If applicable, we recommend that you deposit your laboratory protocols in protocols.io to enhance the reproducibility of your results. Protocols.io assigns your protocol its own identifier (DOI) so that it can be cited independently in the future. For instructions see: <http://journals.plos.org/plosone/s/submission-guidelines#loc-laboratory-protocols>

We look forward to receiving your revised manuscript.

Kind regards,

Abhijit P Pakhare, M.D.
Academic Editor
PLOS ONE

**Additional Editor Comments (if provided):**

Manuscript have been improved from its earlier version. However, some points still needs to be clarified.
How was the waiting time determined?

Response: Dear Prof. Pakhare, thank you for the concern. Health facilities including primary health centers and health posts have a record of waiting time for service. They finally calculate the average waiting time for service in their respective health institution and report it to the primary health care unit head with other reportable indicators. Therefore, we took the average waiting time of each of these health posts as appeared on their official report to the primary health care unit (PHCU).

Whether distance from nearest PHCU was explored and evaluated? Distance is an important accessibility barrier which may affect service uptake.

Response: Thank you for the question. We actually did not measure the distance of the nearest health facility or PHCU. We rather seen the availability of health facility near the health post. This was measured by checking the availability of another health facility within the Kebele (Kebele is the smallest administrative unit in Ethiopia) where the HP is located.

Service output has constraints due to population size. For example, number of ANCs can’t be increased than existing birth rate. So, output has to be scaled or redefined considering population, crude birth rate and expressed in terms of percentage of ANC examination vis a vis expected examinations as per guidelines. Thus, number of ANCs will be driven by their population size. Same logic applies to other health services as well like FP services, diarrheal case, malaria case treatments, child vaccination services, referral services and also household visits. Thus, catchment population seems to be an effect modifier and not only confounder which can be adjusted by multivariate analysis. Consider sub-group analysis by stratifying according to population size

Response: Sure, the size of the catchment population can be an effect modifier to the service outputs. However, we did not use the service outputs as an outcome variable. We used this variable to measure efficiency of health posts. The analysis we employed to assess the technical efficiency of health posts was a non-parametric test, data envelopment analysis (DEA). We used variables like number of health extension workers as input and variables like number of ANC as output to run the DEA. The outcome variable for this analysis is technical efficiency. The size of the catchment population might be an effect modifier to the output rather than the outcome variable (technical efficiency).

**Comments to the Author**

1. If the authors have adequately addressed your comments raised in a previous round of review and you feel that this manuscript is now acceptable for publication, you may indicate that here to bypass the “Comments to the Author” section, enter your conflict of interest statement in the “Confidential to Editor” section, and submit your "Accept" recommendation.

- Reviewer #1: All comments have been addressed
- Reviewer #2: (No Response)
- Reviewer #4: (No Response)

2. Is the manuscript technically sound, and do the data support the conclusions?

The manuscript must describe a technically sound piece of scientific research with data that supports the conclusions. Experiments must have been conducted rigorously, with appropriate controls, replication, and sample sizes. The conclusions must be drawn appropriately based on the data presented.

- Reviewer #1: Yes
- Reviewer #2: Partly
- Reviewer #4: Yes

3. Has the statistical analysis been performed appropriately and rigorously?

- Reviewer #1: I Don't Know
- Reviewer #2: N/A
- Reviewer #4: Yes

4. Have the authors made all data underlying the findings in their manuscript fully available?

The [PLOS Data policy](http://www.plosone.org/static/policies.action#sharing) requires authors to make all data underlying the findings described in their manuscript fully available without restriction, with rare exception (please refer to the Data Availability Statement in the manuscript PDF file). The data should be provided as part of the manuscript or its supporting information, or deposited to a public repository. For example, in addition to summary statistics, the data points behind means, medians and variance measures should be available. If there are restrictions on publicly sharing data—e.g. participant privacy or use of data from a third party—those must be specified.

- Reviewer #1: Yes
- Reviewer #2: Yes
- Reviewer #4: Yes

5. Is the manuscript presented in an intelligible fashion and written in standard English?

PLOS ONE does not copyedit accepted manuscripts, so the language in submitted articles must be clear, correct, and unambiguous. Any typographical or grammatical errors should be corrected at revision, so please note any specific errors here.

- Reviewer #1: Yes
- Reviewer #2: Yes
- Reviewer #4: Yes

6. Review Comments to the Author

Please use the space provided to explain your answers to the questions above. You may also include additional comments for the author, including concerns about dual publication, research ethics, or publication ethics. (Please upload your review as an attachment if it exceeds 20,000 characters)

Reviewer #1: I have reviewed the responses to the comments I made on review of the initial submission. I support the acceptance and publication of this paper in its revised form.

Thank you very much

Reviewer #2: Dear Authors,

Please read again carefully my previous Review. Neither a single ONE out of my recommendations have been adopted. Thus I firmly believe this Manuscript revision is not mature for publishing in its current appearance.

**Response**: Thank you very much for raising these important issues and providing essential literature. Now we have answered your concerns in the revised copy.

Reviewer #4: Overall:

This is an important contribution. After the revisions, this version of the paper presents a clear and consistent analysis.

However, I have 2 major comments which the authors need to address before publication:
1. As indicated in the earlier round of review by Reviewer 1, there is a disconnect between the title and the contents of the manuscript. The title suggests that the assessment is far broader than what the content actually addresses. The title should be revised to reflect this for 2 reasons:
(a) It is about the inefficiency in the HE program, which is captured through an evaluation of the the health posts as far as I can see.
(b) It does not get into any analysis, qualitative or quantitative, of inefficiencies of community health programmes in general.

**Response**: thank you for this relevant concern. Now we modified the title in a way that indicates the whole paper.

2. For interpreting the associations that the econometric exercise throws up, it is very important to be upfront about two things: (a) Caution about establishing causality among variables by making clear the assumptions in using the health posts related data to reflect the HE program, the latter being at a programmatic level as opposed to the former. (b) The limitations or caveats in interpreting the findings as an assessment of the health extension programme and, in general to a community health programme, have to be adequately acknowledged and mentioned in both the introduction and the concluding part of the paper.

**Response**: You are right. In this study we did analysis on health posts and just thought a proxy for health extension program. Based on reviewers’ suggestions and discussion among ourselves, we decided to make the entire focus on health posts. However we have shown the implications of the findings in relation to community health services.
